# Supplementary figures and images for: Estimating receptive fields of simple and complex cells in early visual cortex: A convolutional neural network model with parameterized rectification
Source: PLoS Comput Biol. 2024 May 31;20(5):e1012127. doi: 10.1371/journal.pcbi.1012127 (PMC11168683; doi:10.1371/journal.pcbi.1012127)

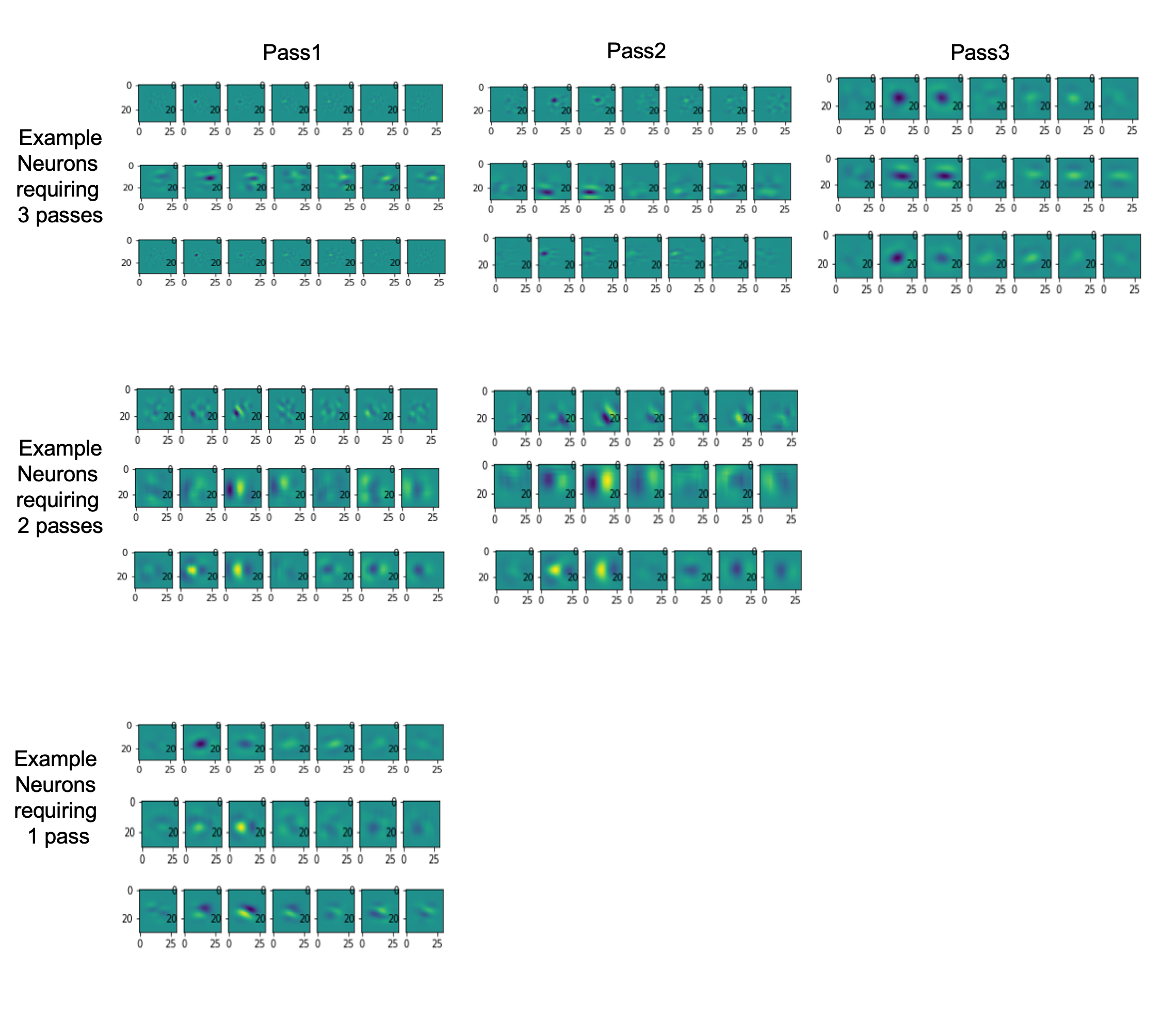

Supplement: S1 Fig — First 3 example neurons require 3 passes and the receptive fields across 3 passes are shown. Second set of three example neurons require only 2 passes and the last set of 3 neurons require only 1 pass. (TIF) [file pcbi.1012127.s001.tif]

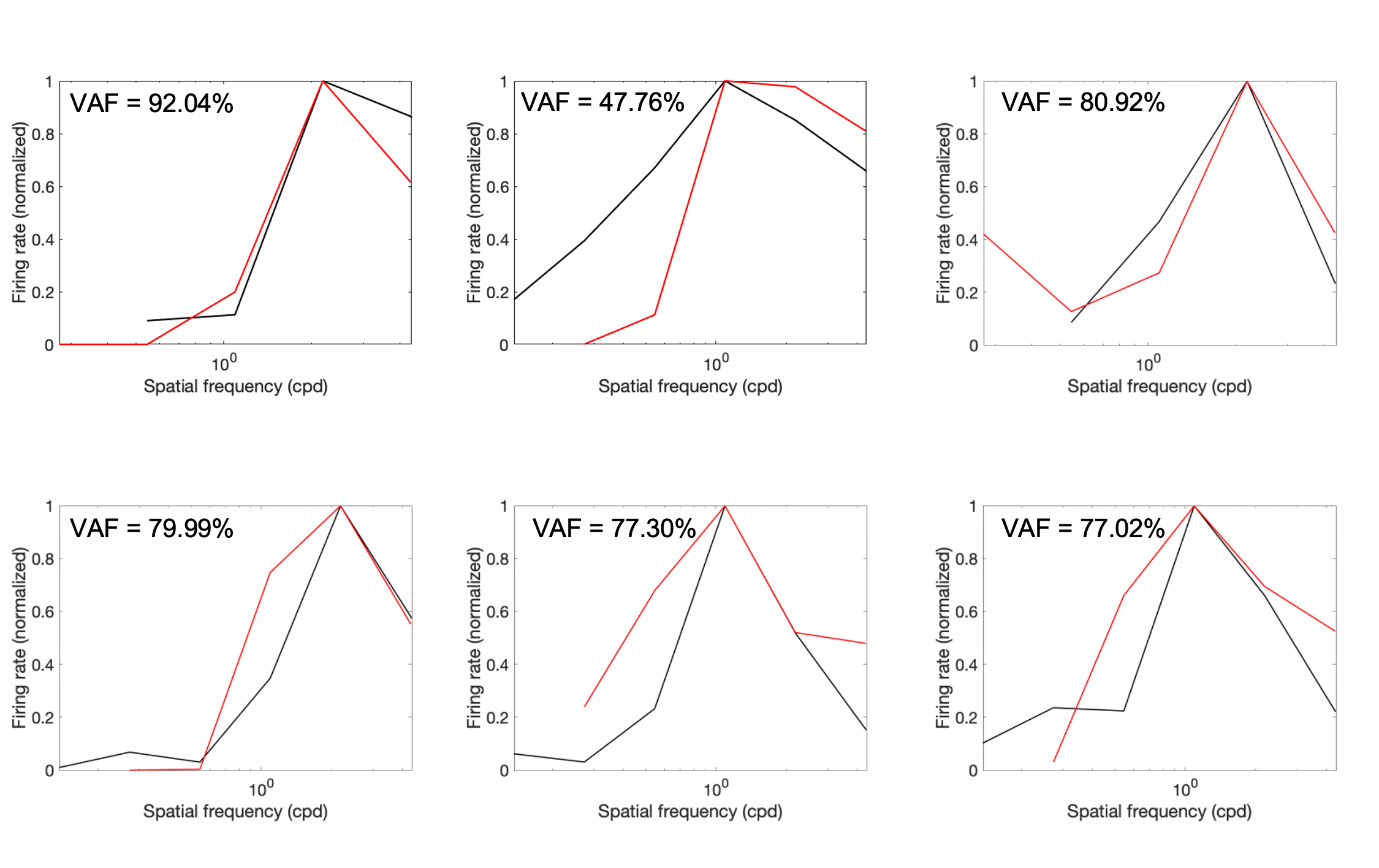

Supplement: S2 Fig — Spatial frequency tuning curve (average firing rate, normalized to maximum) for 6 example A17 neurons. Temporal frequency, 2 Hz; average of 10 repetitions of 1 sec each. Solid black line for neuron’s response to the grating stimuli, red for tuning curve predicted by convolutional model. (TIF) [file pcbi.1012127.s002.tif]

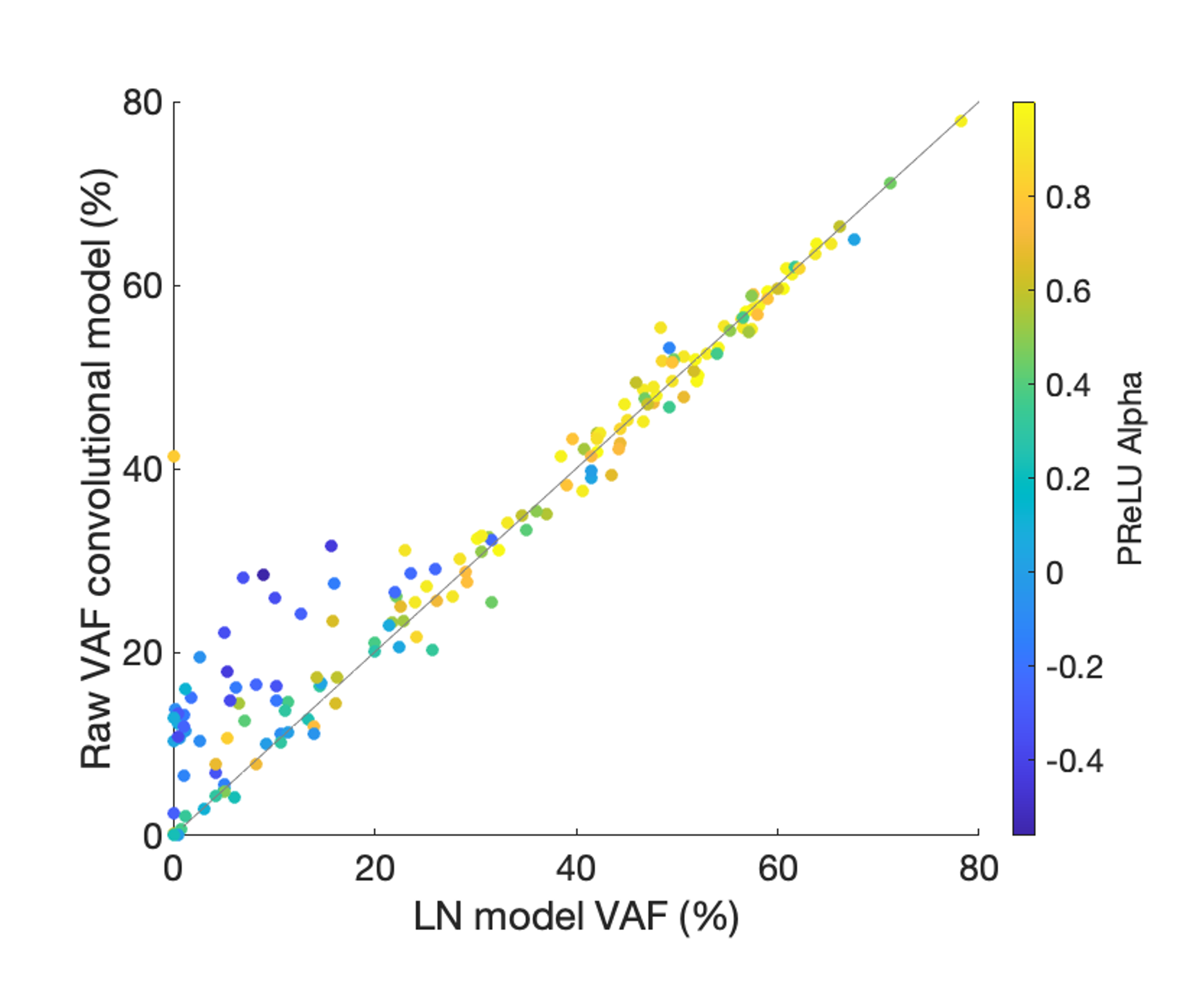

Supplement: S3 Fig — Comparison between predictive performance (test VAF) for the convolutional model and a simpler linear-nonlinear (LN) model. Each point denotes a neuron, color coded according to its estimated PReLU α parameter. (TIF) [file pcbi.1012127.s003.tif]

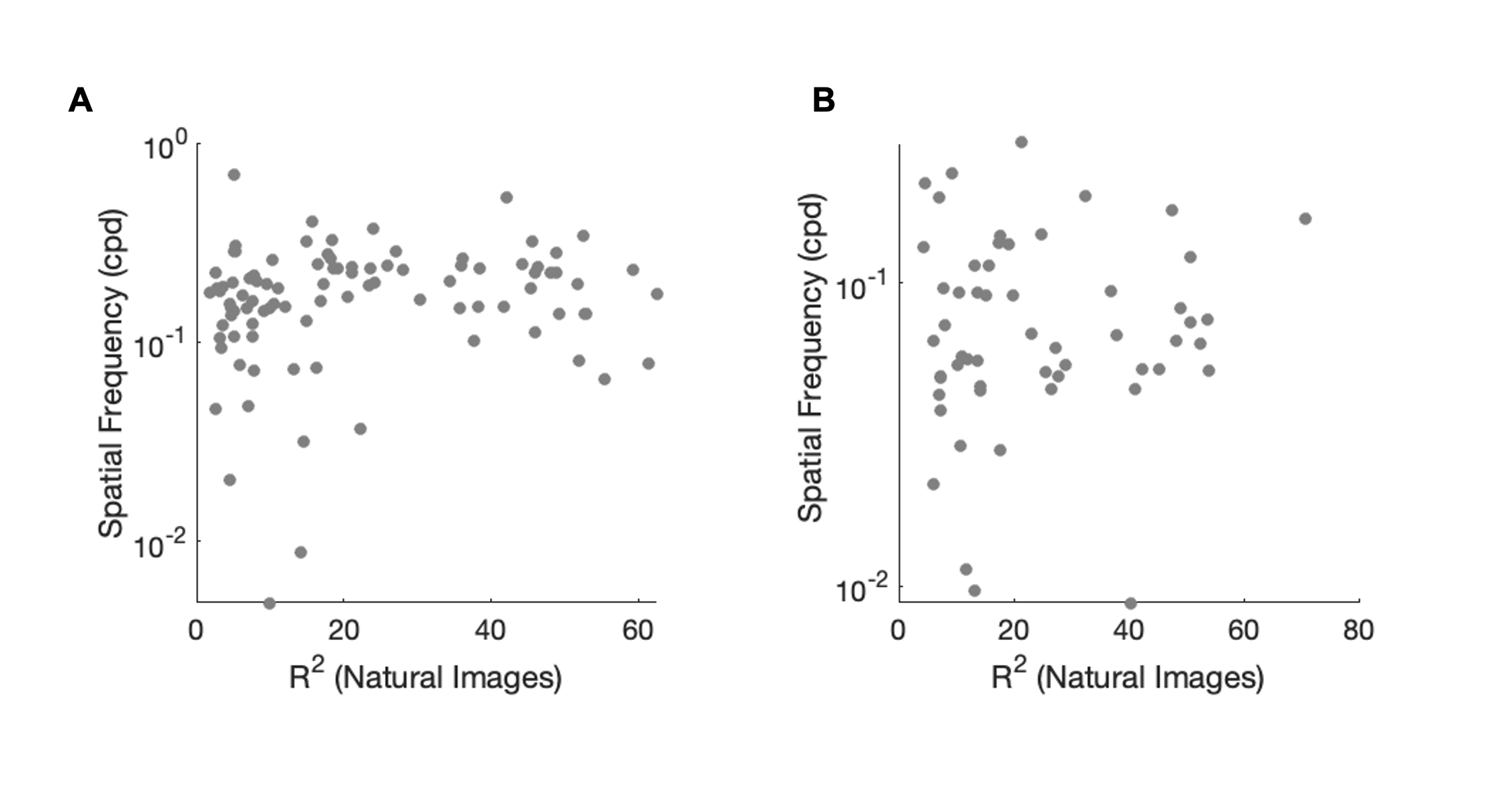

Supplement: S4 Fig — A) scatterplot of optimal spatial frequencies vs reliability ratio (R2) values for neurons in the A17 sample. B) Similar to A, for A18 neurons. (TIF) [file pcbi.1012127.s004.tif]

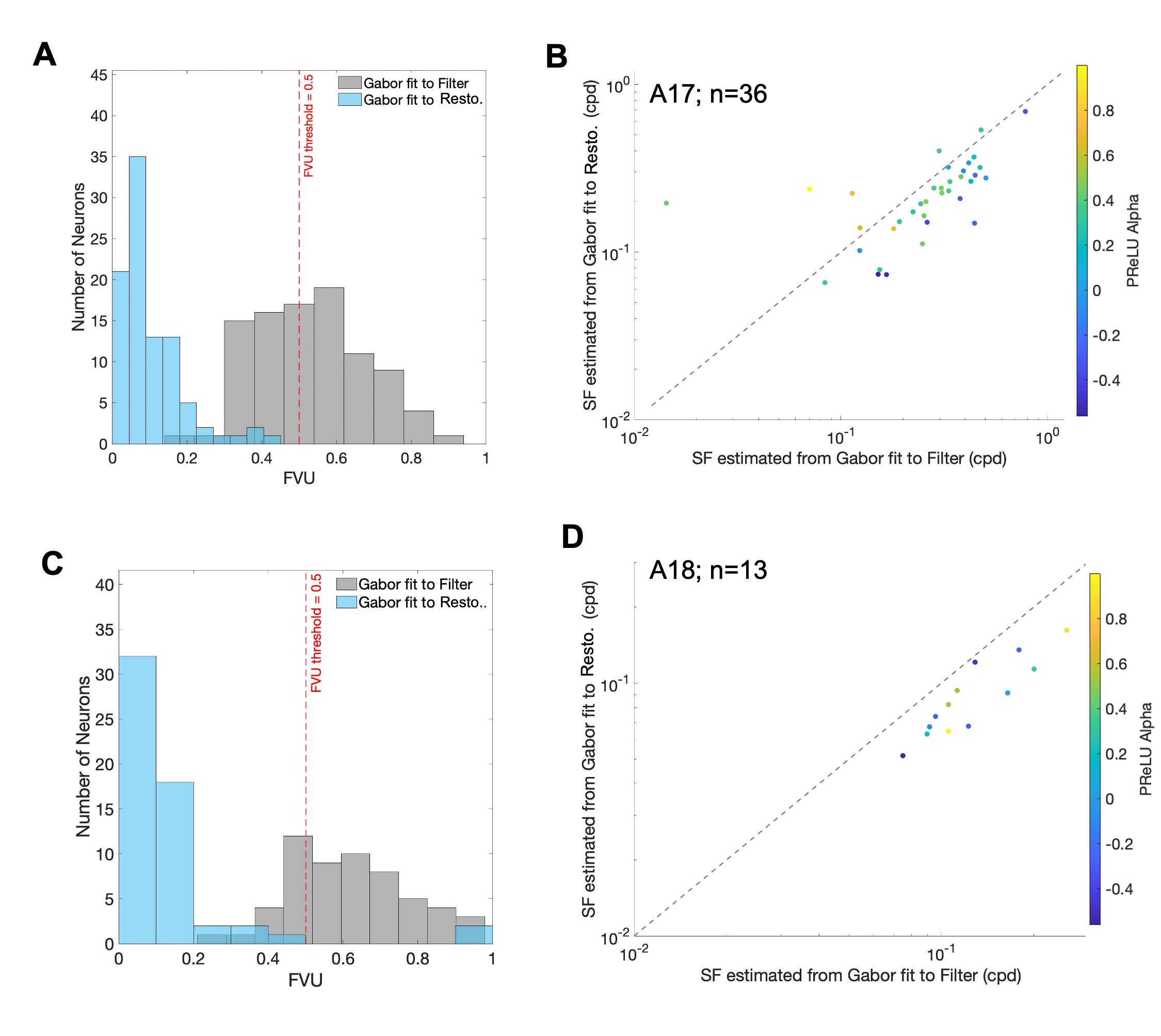

Supplement: S5 Fig — A) Histogram of FVU (fraction of variance unaccounted) values, for A17 sample when Gabor functions were fit to the filter (grey) and restoration (blue). B) Scatter plot of optimal spatial frequency estimated when Gabor was fit to filter vs restoration—each point represents one neuron, color coded according to its PReLU alpha value. C) Similar to A for A18 neurons. D) Similar to B for A18 neurons. (TIF) [file pcbi.1012127.s005.tif]

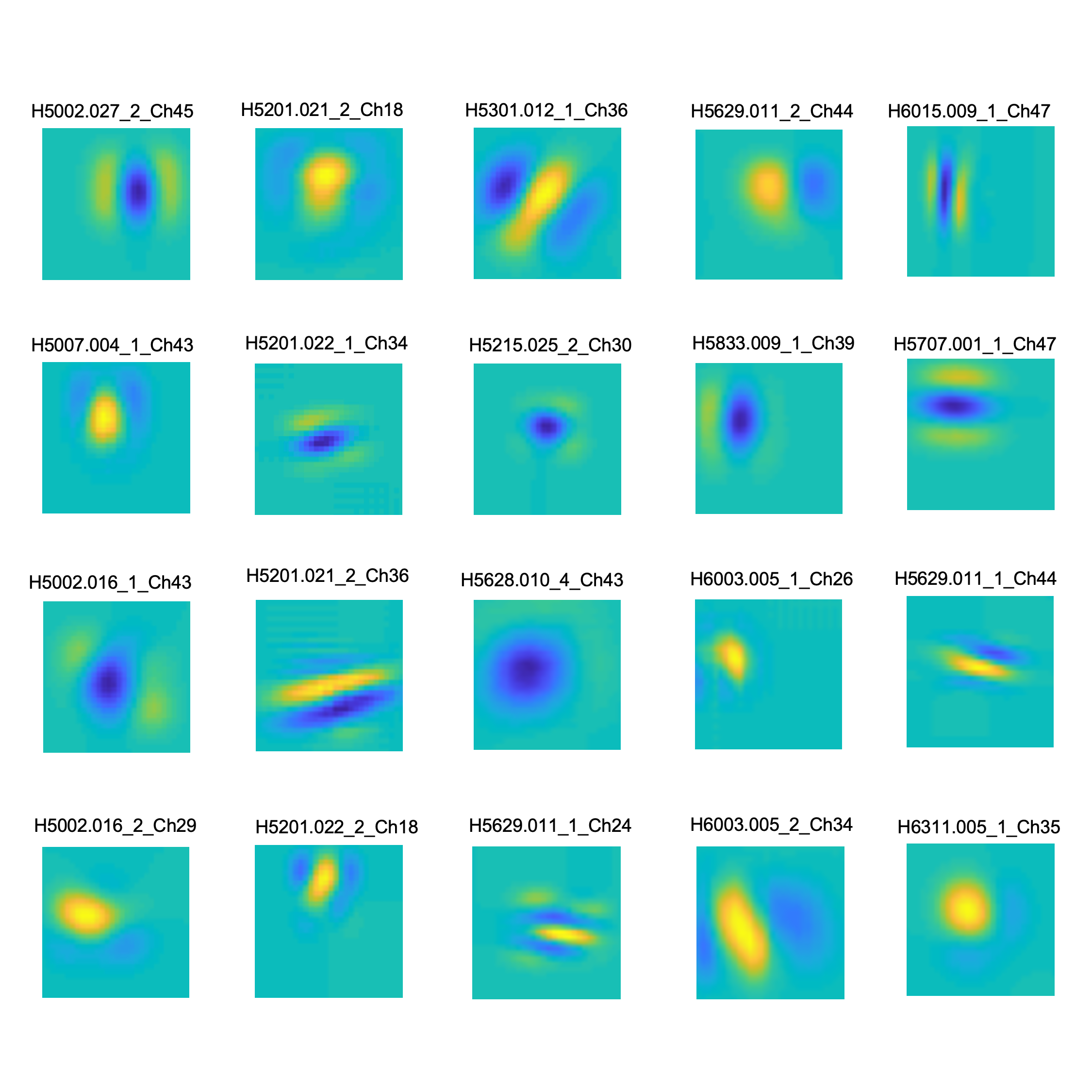

Supplement: S6 Fig — (TIF) [file pcbi.1012127.s006.tif]
