# Supplementary material for: Estimating receptive fields of simple and complex cells in early visual cortex: A convolutional neural network model with parameterized rectification
Source: PLoS Comput Biol. 2024 May 31;20(5):e1012127. doi: 10.1371/journal.pcbi.1012127 (PMC11168683; doi:10.1371/journal.pcbi.1012127)
Supplement: Attachment — Submitted filename: Author Replies to Reviews.docx [file pcbi.1012127.s007.docx]

**Reviewer 1:**

1. The use of “reconstruction” – convolving the summation layer with the convolutional layer – to smooth and visualize tuning is interesting. In the case of a purely simple cell (alpha=1), it makes sense (linear+linear = linear). However, for a complex cell, say with alpha=0, it seems like the convolution would have the effect of smoothing out high spatial frequency tuning that might be present in the initial convolutional layer, thus biasing estimates of spatial frequency tuning to lower values. Perhaps this concern was addressed in the manuscript, so it’d be great if the authors could clarify if this is/is not a concern. Alternatively, is it possible to fit the Gabor to the convolutional layer alone (without “reconstruction,” for some neurons with high SNR, perhaps) to test for possible bias in measurements of tuning for complex cells.
   Lesser, but related, the term “reconstruction” is potentially confusing to readers. Previously this term has been used to describe decoding analysis where stimuli are reconstructed from neural population activity. The authors might consider a different term.

This is a good point. Using model simulations, we confirmed that fitting Gabor functions to a linear reconstruction can bias the spatial frequency estimates to lower values - however for realistic models, i.e. RFs encompassing very few cycles of the optimal filter SF, this effect was minor, for both simple and complex cells. When we performed similar Gabor fits to the filter estimates (not withstanding their higher variance and lower FVU values), the estimated optimal SFs were highly correlated, with the those from the reconstructions being only slightly lower. Consequently, we used the linear reconstruction estimates for further analysis and comparisons, due to their greater reliability as shown by significantly lower FVU values for Gabor fits. To address this matter, we have added a paragraph explaining and acknowledging this issue on page 42 and supplementary figure 5.

In the manuscript we have changed the term “reconstruction” to “restoration” to avoid confusion.

2. Is there a concern that variations in explainable variance (R^2_neuron) could impact measurements of tuning properties? For example, if a unit is overall noisier, will the AC/DC ratio trend toward lower values? It’s great that the authors consider noise ceilings in their analysis, but it seems important that they also determine if any differences in tuning (e.g., complex vs simple cells, A17 vs. A18) is robust to differences in response reliability. One idea for addressing this would be to degrade high-R^2_neuron spike trains to values that match noisier neurons and show that the reported differences are preserved when response SNR is matched. There are likely to be alternative controls that accomplish the same thing, but the authors should consider how response reliability can impact measures of tuning.

We tested whether the estimated spatial frequencies depend on the R^2 of neurons in both A17 and A18. The analysis didn’t show any correlation between the estimated spatial frequencies and R2. We have now documented this on page 42, and in supplementary figure 4.

It is conceivable that the AC/DC ratio measured from grating responses might be biased to lower values (more complex-like) by greater noise. However, it seems unlikely that a similar effect would be evident for the PReLU alpha measured with our system identification. We have now mentioned this in the Discussion section, on page 49.

3. The model appears to perform reasonably well, in terms of variance explained. However, someone considering use of this approach would want to know if it is really more effective than alternative, perhaps more established encoding models. It may not be practical to perform an exhaustive comparison of all possible alternatives. But is it possible to fit a traditional LN model and compare performance? A very quick and dirty LN model would be to freeze alpha at 1 and fit the other parameters. Or, is there published data that has been fit with a different model that could be run through the current code? Some kind of quantitative comparison would be informative.

This is a good point and to address this, we compared the model performance to a simple LN model. The results showed that our model works better, particularly for complex cells. These results are now discussed on page 37 and in supplementary figure 3.

LESSER
p. 2 “compensation for this unreliability” This phrasing is confusing. The term “noise ceiling” is reasonably well established and might be useful to frame this result. (as in the author summary)

Thank you for pointing this out. We have reworded this (bottom of page 2).

p. 6-7. The authors might note early work by Prenger at al. (Neural Networks 2004) that fit full spatio-temporal models using ANNs.

Thank you for pointing this out. We have now noted the Prenger at al. study and its possible limitations (pages 6-7).

p. 11. Can the authors report the number of distinct images that were presented in each subset (training/regularization/testing). 20 x 375 = 7500 unique images. Is this correct? Were the stimuli and groupings identical for all neurons

We used 20 training movies resulting in 20 x 375 = 7500 unique images and 5 regularization/testing movies resulting 5 x 375 = 1875 unique images. We have now included these details on page 11.

p. 11-14. It’s helpful that the authors identify free parameters and point out their relatively low number compared, e.g., to CNN models. Can they report the range of total free parameters required for the complete model? It does appear to vary depending on the size of the convolutional filter. Is that the only source of variation?

Yes, the number of free parameters depend on the size of the filter. When the filter size is 11, we have a total of 855 model parameters (filter: 11 x 11 x 7 + PReLU: 1 + Map layer: 5 + Output nonlinearity: 2) and when the filter size is 15, its 1583 parameters (filter: 15 x 15 x 7 + PReLU: 1 + Map layer: 5 + Output nonlinearity: 2). We have now discussed this on page 17.

p. 16. Was any regularization imposed on the model fits? An L2 penalty for the convolutional layer would probably not improve performance substantially, but it might clean up the fits.

In addition to early stopping regularization (with a patience parameter of 50), we did impose L2 regularization for the conv2D layer, using its default value of 0.01. Preliminary analysis with a subset of neurons revealed excessive smoothing (estimated filters appeared blurred) of estimated filters and decrease in VAF accuracy when the L2 value was increased to values more than 0.01. Therefore, we kept the L2 value fixed at 0.01. We have added an explanation of this on page 17.

p. 17. R^2_neuron. Is there a reference for this approach to measuring the noise ceiling? It appears that he 19-rep PSTH is treated as a sort of ground truth response against which a noisy response is compared, but can’t the 19-rep PSTH still be noisy if the unit has a very unreliable response. Maybe the logic just needs to be spelled out more clearly.

The “noise ceiling” calculation approach used in our study is similar to the “oracle score” defined in, e.g., the Vintch et al and Walker et al studies. The idea is that the x19 averaging would greatly reduce the noise, though not totally eliminate it. However, it can serve as an estimate of the ground truth. We have added this explanation on page 18-19.

p. 19. “time lag of maximal variance” Were the linear filters typically space-time separable? If so, one could possibly improve spatial tuning estimates by computing the first principal component of the filter in space vs. time.

This is a good point. Model performance could presumably be improved by using PCA if the neuronal receptive fields were space-time separable. But cat A17 and A18 have a substantial portion of space-time non-separable neurons. We have included this explanation on page 20.

p. 23. Fig. 2C. Can the authors superimpose the individual data points in this box plot? Same request for some of the later box plots.

We superimposed the data points on each box plot (now Fig 2D, page 25)

It would also be helpful if the authors could explain what “linear reconstruction” (or some alternative term, see above) means when they plot it in Fig 2.

We have included this in figure 2 legend (page 25).

p. 27. Fig. 5A. Can the authors provide a similar plot showing noise-corrected model performance?

This is a good point. We have plotted the noise-corrected VAF (i.e. explainable VAF) values in figure 5A (page 30).

p. 35. “FVU” is this 1-VAF/100? Is there a reason for switching to a different error metric here?

Yes - we have defined FVU in this way to be consistent with Ringach et al and also to avoid possible confusion with VAF predictive performance. We have now explained this (page 22, top).

p. 44. The use of PReLU is a nice innovation. It is slightly confusing, though, in if/how it would deal with a cell that is, say the sum of three simple cells in quadrature phase (ie, a sum of simple cells that is not purely complex). Is this a limitation of the PReLU approach vs. a subunit model?

Yes, a caveat regarding our model will be the inadequacy to capture multiple kinds of subunits. Possible solutions might be the "NIM" (McFarland et al), which is already discussed at top of page 54) or by adding additional filters, though these approaches would be at the expense of many additional model parameters. We have added new text about this at the top of page 54.

p. 48. The authors might note that David et al 2004 specifically observed that temporal frequency tuning differed between stimulus with natural and whitened temporal dynamics, possibly reflecting adaptation to stimuli that do not change rapidly.

This is a good point. We have now included this in the "Temporal dynamics" part of the Discussion (near top of page 53).

TYPO/GRAMMAR
p. 4 “However, predicting responses … through system identification [3].” There’s something funny about this sentence.

We have re-worded this sentence.

p. 27. Fig. 5 legend. “holdback”. Should it be “held-out”?

Actually, we are pretty sure that current usage is "holdback" - certainly that's what a google search shows.

p. 44. “it’s opposite” should be “its opposite” - now page 48 ...

We have corrected this.

**Reviewer 2:**

Reviewer #2: Nguyen et al. used a convolutional model with parameterized rectification to estimate receptive field properties of simple and complex cells in cat areas 17 and 18. They found that the convolutional model could predict the responses of simple cells better than those of complex cells to both natural images and drifting gratings. The poor predictive performance of complex cells was primarily due to low trial-by-trial reliability. Moreover, they found that the parameterized rectified linear unit (PReLU) value correlated well with the AC/DC ratio, a traditional measurement for simple/complex segregation. However, the distribution of the PReLU values was not bimodal, indicating that simple and complex are not discrete cell types. The authors also showed that the receptive field properties measured with natural images are comparable to those with artificial images (such as sparse noise and drifting gratings) in cat areas 17 and 18.

Overall, the authors provide a straightforward model that can capture many features of simple and complex cells. The methods are described in detail, and the discussion appropriately covers most of the findings. However, a couple of issues need to be sufficiently addressed.

Major:
1. Some important terms and claims need to be addressed appropriately. Please provide additional statements and explanations for the following points.
-- The authors did not specify how the filter layer and its size were determined. The method used to define the filter layer is critical because it determines the features of simple and complex cells, including the number of subunits, preferred orientation, spatial frequency, etc. What’s the procedure for the optimization? This question is critical because the choice of window size largely affects the fitting wellness and the VAF values.

This is a good point. The choice of the filter size affects the model performance. For each neuron, we evaluated window sizes of 11 and 15 and chose the one giving the highest predictive accuracy VAF while also providing minimal clipping at the edges. We have added a sentence about this on page 16.

-- The authors claimed that the relative difference in the VAF between complex and simple cells was lower by eliminating the trail-by-trail unreliability of complex cells. Please provide data to support the claim

Thank you for pointing out this omission. After adjusting for the noise ceiling of each neuron, the explainable VAFs were less different between simple vs. complex cells. We have now added explainable VAF results in both panels of Figure 5, including simple vs complex cells in panel B, with discussion on page 29 (bottom of page).

-- The trial-by-trail reliability for simple and complex cells is much higher for natural image responses than drifting gratings responses. Please provide explanations for the difference.

The observed changes in the trial-to-trial response variabilities could be due to the natural image movie’s temporally abrupt changes, which might facilitate response synchronization compared to the smooth motion of gratings as shown by Baudot et al, 2013 also. We have now included this explanation on page 34.

2. There are many discrepancies in receptive field properties, such as preferred spatial frequency and aspect ratio (nx and ny), between measurements in the study and those in the literature. The contradicting results are probably due to the difference in stimuli used for receptive field mapping: the authors used natural images, whereas other pieces of literature used sparse noise. The authors should recheck the data, explain these discrepancies in the discussion, and include more cat literature on spatial features. Here are the details.
-- Fig.11A. The preferred spatial frequency in most area 17 neurons is less than 0.4 cpd. In contrast, Movshon et al. (1978) reported a 0.3-3.0 cpd range.

This is a good point. The observed differences in SF preferences could be due to the different tested eccentricities of the receptive fields. We have pointed this out on page 41 (bottom).

-- Fig. 12. Most of the data points in the distribution of nx and ny are below the unity (1:1 ratio) line, but Jones and Palmer (1987, studying cat simple-cell receptive fields, Table 1, replotted in Ringach 2002) reported the opposite – most of the data points are above the

unity line.

Thank you for pointing out this discrepancy. The observed differences in nx/ny from our results, as well as those of Ringach (2007), could conceivably be due to differences in the stimuli - i.e. the 2D sparse white noise of Jones and Palmer used 2D white noise.

What all these results agree on is that at least for simple-type cells, the nx-ny plots largely fall along a one-dimensional continuum, implying that the Gabor-like filters are subject to an underlying constraint. Filters produced by unsupervised learning methods applied to natural images (Ringach 2002; Rehn & Sommer, 2007) also indicate similar one-dimensional loci, suggesting that this constraint is related to efficient coding of natural scenes.

We have added two paragraphs about these points, on page 52.

Minor:
1. Page 10. Drifting gratings were used to measure the AC/DC ratio and spatial frequency tuning. Please specify the optimized grating (i.e., size, contrast, orientation, temporal frequency).

We optimized grating orientation and temporal frequency specifically for each neuron, and we used a fixed contrast for all neurons. We have clarified this on page 11.

2. Page 22. The authors used “R2neuron < 1% and explainable VAF < 1%” to exclude neurons with insufficient response reliability. What’s the rationale behind these criteria because one percent seems to be very low?

We chose these values because we noted that even for these lower VAF and R^2^_neuron_ values we observed well-structured receptive fields. We have clarified this on page 23.

3. Page 23-26. In Figs. 2-4, the window size of the linear reconstruction is twice as large as that of the filter. If the purpose of these figures is to represent the similarity of the maps increase with the cropping procedure, it would be better if the two window sizes were the same. Furthermore, the scale bar is marked with only “+” and “-“. Is the map normalized by dividing the absolute maximum value of the map?

The main purpose in Fig 2A is to show how improved cropping windows give RF estimates with clearer spatial structure, for both the filters and the reconstructions. The linear reconstruction dimensions are 30x30, which results from convolution of a 15x15 filter with the 16x16 gaussian map - thus the reconstruction is twice as large as the filter itself. Yes, the figure panels depict normalized values and hence the scale bar “+” and “-”. We have added more explanation of this in the legend for Figure 2 (page 25).

4. Page 28. In Fig. 6, I suggest the authors add a third figure representing the comparison between simple and complex cells (just like Fig. 5B) because that’s one of the main themes in the manuscript.

We have included a subpanel C in figure 6 (page 31) to show these results.

5. Page 47. The discussion regarding the aspect ratio of spatial receptive fields should include the literature on cat areas 17 and 18. Receptive field properties in cat and monkey V1 are similar but not identical. For example, the receptive field size is smaller, and the preferred spatial frequency is higher in monkey V1 than cat V1.

Note that the nx and ny values are dimensionless, and are implicitly normalized for spatial frequency, as can be seen from Eq 8 - as stated in the text (page 44), they reflect bandwidths of SF and orientation tuning.

Yes, we agree with the species differences. The main study we referred to here, Ringach (2002), also includes a comparison of data from both macaque and cats (his Fig 5). However, our results might deviate from theirs due to differences in stimuli. We have added some discussion of this on page 52.

**Reviewer 3:**

Reviewer #3: In this manuscript, the authors develop a simple convolutional neural network model approach to estimate spatio-temporal receptive fields in A17/18 of the anesthetized cat by predicting responses to natural images. The most interesting aspect of the model in my view is that its parameters can be related to biological properties of visual cortical neurons in relatively straightforward ways. In addition, the model is able to recover both simple and complex cell RFs, and predicts a continuum along this dimension, as previously found with data-driven measures. The authors then use the model to compare the fitted RFs to known aspects of cat visual cortex neurons and to predict responses to novel stimuli not used for model fitting. Overall, the authors find that the RF properties estimated with the novel model match well the expectations from the literature.

The manuscript is well written and will be interesting to a broad range of scientists working on selectivity in sensory systems and novel modelling approaches to capture those. In addition, the manuscript might serve as an important link between the novel modelling approaches applied most often nowadays to data from mouse V1 to a substantial and important body of work from the visual cortex in cats.

MAJOR:
1.) To which degree is the general similarity between the fitted RFs and RFs in other studies of cat visual cortex a consequence of the fitted model? In other words, to which degree would the novel model be able to account for some of the more unconventional RFs that have recently been reported in mouse V1 (e.g. Cadena et al. 2019)? Maybe these unconventional selectivities do not exist in the cat or under anaesthesia, but it would be important to understand what would be possible to recover by the model if RFs were unconventional, such as in Fig. 3 of Cadena et al. 2019.

Our system identification does not involve any prior on the shape of the RF, so the estimated RFs could potentially take any shape. The Gabor-fitting is applied only afterwards, and indeed some estimated RFs do depart noticeably from classic oriented Gabor-like functions, e.g. non-oriented, crescent-shaped, etc. We have now shown several examples of estimated receptive fields in Supplementary Figure 6, and we have discussed this on page 55-56.

We did not notice receptive fields in the Fig. 3 of Cadena et al. 2019 (which is based on data from awake macaques). However, mouse V1 receptive fields, e.g. Fig 11 in Niell and Stryker, 2008 (<https://www.ncbi.nlm.nih.gov/pmc/articles/PMC3040721/>) shows examples of spatial receptive fields with one, two, or three subfields, and with varying orientation, ON/OFF centers, and spacing of subfields. Our model is also capable of capturing these variations in receptive field shapes, as discussed in the above paragraph.

2.) One important step seems to be the “cropping” of the stimulus images in the three-pass procedure. Could the authors please provide quantitative data that 3 passes are enough and optimal, i.e. does the fit not improve for more passes? Are three passes warranted etc? They also mention that the majority of A18 / a subset of A17 neurons had large RFs and only required 1 or 2 passes - could the authors please provide some illustration or quantitative assessment regarding which neuron is fit with how many passes and why?

VAF improvements after pass 3 were diminishing, and we therefore decided to not pursue further passes. The final pass was qualitatively assessed to insure it captured the receptive field fully without clipping at the edges. We have added further explanation about this on page 24. We have shown some example neurons with their final pass as pass 1, pass 2 and pass 3 in Supplementary Figure 1.

3.) To improve the clarity of the description, would it be possible (and make sense) to indicate the spatial relationships in the RF figures, e.g. Fig. 2? I see that through each pass, both the filter, linear reconstruction, and the map have a smaller and smaller spatial scale, but I would find it easier to understand if I saw which portion of the inputs are chosen for the subsequent passes, and how filter and reconstruction and map are spatially overlaid.

This is a good point. We have shown the cropping windows the example neuron of Figure 2, overlaid on an example natural image stimulus, in Figure 2C.

4.) As mentioned above, I believe the study has great potential to serve as link between the newer results on V1 selectivity in mice and the broad literature from cats. To exploit that potential, I would encourage the authors to relate to the new literature, at least in the discussion to bring up species differences beyond macaque / cat, issues of anaesthesia / wakefulness / freely moving, etc. that might explain differences and similarities. I understand that there is no direct comparison possible and this would be somewhat speculative, but I believe this would widen the scope of the paper and potentially the readership and applications of the method.

Our system identification model architecture should be applicable to map receptive fields at early stages of visual processing in any species, provided they do not have multiple types of subunits, or require models with more linear-nonlinear stages (i.e. deeper neural network models). For example, our approach should be able to capture receptive fields, including ones that are not "Gabor-like", measured by reverse-correlation or by spike-triggered covariance, e.g. Fig 11 in Niell and Stryker, 2008.

An important finding with recordings from awake, behaving mice, has been that V1 neurons can respond not only to visual stimuli, but also exhibit responses related to locomotion (Niell and Stryker, 2010) or other spontaneous behaviors (Stringer et al 2019). Our model obviously does not incorporate such motor-related responses, but it could be extended with the addition of regressors against signals related to such behaviours. We have added two paragraphs about all this, on page 56.

5.) The authors separately sort the different experiments and post-hoc align clusters. What was the motivation for this choice? Would it not be better to concatenate the data from both experiments and sort only once? I understand that it is very labour-intense to optimize this step, but could the authors please at least provide quantitative evidence that the alignment procedure works as intended? What is “high confidence”?

This approach was chosen because of concern over the possibility of slight slippage of the probe through the brain tissue, causing changes in spike amplitudes, more so than in waveshapes - especially a concern here since natural image and grating responses were often collected at different, non-contiguous times. We have added explanation of this on page 15.

6.) p. 23: What do the authors mean by “contained a central inhibitory region and surrounding excitation”? Do the authors want to say that bright stimuli decreased the responses in the center and increased the responses in the surround? Or that dark stimuli in the center increased the response?

Yes, that would be the prediction for the estimated model.

7.) For the comparison of measured and predicted SF tuning, is it typical to have systematically different shapes of the tuning curves although the peaks align (as for the example in Fig. 6A)?

We did not attempt any detailed assessment of prediction of the shapes of SF responses, just the VAFs. However, from comparisons of multiple examples, a general statement about this seems unwarranted. We have now shown several examples in Supplementary Figure 2.

MINOR: We have corrected these.
- p. 5: (for example [9, 10]. -> add missing closing bracket
- p. 9: (ver. 3.0.10; [31, 32] -> add missing closing bracket
- p. 35: (e.g., [29, 46, 47], -> add missing closing bracket
